# Some Account of the Symptoms and Treatment of the Dengue as It Appeared and Prevailed in the Southern and Central Parts of Ohio, in the Spring of 1829

**Published:** 1829

**Authors:** John Cook Bennett

**Affiliations:** McConnelsville


Art. V.—*Some account of the Symptoms aud Treatment of the Dengue, as it appeared and prevailed in the southern and central parts of Ohio, in the spring of* 1829. By Dr. John Cook Bennett, of McConnelsville.

During the spring of the present year, 1829, the Dengue prevailed in Circleville, Marietta, McConnelsville, Malta and other towns lying in the valleys of the Scioto and Muskingum rivers. My own observations on this new visitor were chiefly made at the first of those towns.

### Symptoms.

The symptoms of Dengue, as it appeared in Circleville during the months of March, April, May, and June, A. D. 1829, were much milder than those of the south. This may be attributed to its having changed its latitude. The disease commenced with a chill, which continued from half an hour to an hour and a half, succeeded by fever which continued from one to four days before the cutaneous efflorescence made its appearance. The patient complained of lassitude, and an uneasy sensation in the limbs, pain in the back, head, and extremities. The pain in the head was an acute cephalalgy. It would be well to remark, that although there was more or less pain in the joints, there was no observable swelling.—There was some itching of the skin. The eruption or efflorescence differed essentially from that of measles, which prevailed at the same time. The eruption in measles is flattened, that of the Dengue, or "French measles" as it was called, was elevated with an inflamed base, and pointed in the centre, not unlike prickley-heat. The eruption in measles, in many cases, was confluent, that of the Dengue was in all cases distinct; though it often appeared in clusters, blotches, or wheals, on separate parts of the body. The eruption in measles was general, that of the Dengue was principally confined to the thorax, neck, and face. In some cases a few pimples appeared upon the extremities. In a word the Dengue eruption may be said to range between the miliary eruption and that of measles, as to the size of the pimples, but it is rather more pointed than either. The eruption continued upon the surface from one to eight or nine days, usually about four, before it totally disappeared. Desquamation of the cuticle attended upon the subsidence of the eruption. The eyes were not generally inflamed, and there was but little or no cough. In this it differed from measles. The adnata was slightly tinged with red vessels. The patient experienced considerable pain when the eyes were moved in their orbits, and they were quite sensible to the touch. There was some soreness of the joints for several days after the eruption had disappeared. In two cases there were relapses after an interval of two or three days. These cases were said to be measles by the attending physician, but I was convinced from the appearance of the eruption, the pain in the joints, the pain in the head, back, and extremities, the absence of cough and sore eyes, &c. that they were Dengue. Some persons supposed that the cases, generally, which were not measles, were miliary fever, and I was of the same opinion until I made an accurate examination of all the symptoms of the three diseases—Dengue, measles, and miliary fever. The tongue was generally red on its margin, but foul in its centre. In some cases the urine was copious, clear and without deposit, in others scanty and high coloured, and in others turbid. Infiltration of the lymphatic glands of the neck and axilla now and then occurred. Many cases were attended with vertigo, and some with great mental uneasiness. Inappetency, and a vitiated taste attended upon almost every case.

The following are the symptoms of the epidemic at Marietta as detailed to me by Dr. Hildreth, in a recent conversation. It prevailed more or less during the months of February, March, and April, and was a disease similar to that described by the southern writers. The symptoms were all milder than those at the south, but of the same character. High fever, preceded by chilliness, continuing for one, two, or three days before the eruption made its appearance, and subsiding gradually as that disappeared. The eruption was chiefly confined to the neck, chest, and body—composed of small red pimples attended with great heat and itching; more diffused than the measles, and drying up in less time. No redness of the eyes, or cough. Severe pain in the joints of the wrists and ancles, ending in swelling and tenderness of the integuments.

Dr. Johnson, of M'Connelsville, informed me that two cases of this disease, attended with mild symptoms, came under his notice. Dr. Barker, of the same place, (McConnelsville) says that no cases have come under his immediate care, but he says that a disease called the "French measles," has prevailed to a considerable extent in the country. Dr. Martin of this county, (Morgan) says that a disease embracing the symptoms of Dengue has prevailed to a considerable extent with him. In several cases there were relapses after an interval of from four to six days. Dr. Martin seems to be of opinion that the disease is a variety of measles, although he says the eruption was more elevated and more distinct. Mr. Dana, near Waterford, Washington County, informed me that the disease prevailed to a very considerable extent amongst the inhabitants. I am informed that Dr. Bowen of Waterford, pronounced the disease to be nettle rash.

The symptoms of Dengue, as detailed by various writers, show that they differ very widely in different parts of the country, though the disease is essentially and identically the same every where. We shall proceed from the symptomatology of this disease, to the methodus medendi.

### Treatment.

The early symptoms of their complaint required an active treatment from the practitioner. In most cases the inflammatory symptoms required that the lancet should be dipped in the *crimson fountain*, and a considerable quantity of blood abstracted. This practice was attended with the happiest consequences in my practice. Dr. Hildreth used the lancet in Marietta with good success. It is disapproved of, however, by Dr. Dickson, and by some of the other southern practitioners. The climate might make the lancet inadmissable in the southern states, when it could be used with propriety and advantage here. Dr. Drake in speaking of the treatment of Dengue says, "It should be borne in mind, however, that in changing its latitude, it may change its character so far as to require a treatment considerably different. We should expect, indeed, that it would assume a more inflammatory nature, and call for a bolder use of the lancet, than it seems to have required in the south." In a communication from Dr. Stennett, of Jamaica, to Dr. Betton, of Germantown, he says, "I find copious bleeding, at first, in able people, followed by small doses of epsom salts and tartar emetic, so as to produce slight nausea and action of the bowels, together with warm salt water baths, generally remove the disease in three days." As a cathartic I generally used flor. sulph and crem. tartar. aa ʒi. pro re nata. In some cases, however, I used calomel in large doses. Pediluvium and hot fomentations were resorted to when we desired to promote natural perspiration. When the pain seemed to demand it, the tinct. opii was used freely and repeated at proper intervals. I frequently directed hot saffron tea as a diaphoretic. This was found to be an excellent remedy. It appeared to relieve all the unpleasant symptoms. Dr. Squaer, who was a great advocate for the use of cathartics in this malady, used the following:—"o℞. extract colocynthidis comp. aloes socoterin. gummi resinæ, aa gr. jjj. hydrargyri submuriatis gr. xxiv. fiat massa in pilula duodecim dividend sumatur tres h. s." Upon the following day the pills were assisted by an infusion of senna or supertartrate of potash.

Some practitioners used cold bathing to the head in cases of severe pain, with good success. Injections with some gentle purgative were of service. The diet was light, and in fact the general regimen was purely antiphlogistic. Warm flannels were often applied to the abdomen in order to relieve urgent symptoms. The southern physicians used vesicants to relieve the sickness of the stomach. The means of cure adopted by Dr. Munos, and other physicians of the Island of Cuba were diet, partial baths of the feet and arms, sinapisms, laxatives, glysters, diluents, topical bleeding, oily and opiate frictions, applied to the painful parts, anodyne cataplasms, fomentations, and lotions of the same class, emollients, &c. &c. The treatment detailed above, succeeded in every case in this climate, so far as I can learn. Some persons did not require the aid of medicine, and were not even confined to bed at all, while others were very sick. I am of opinion that our mode of treatment will coincide with the views of our southern brethren, except so far as it regards blood-letting.

